# Lung bronchiectasisas a paradigm of the interplay between infection and colonization on plastic modulation of the pre-metastatic niche

**DOI:** 10.3389/fonc.2024.1480777

**Published:** 2024-10-14

**Authors:** Lucrezia Pisanu, Klodjana Mucaj, Valentina Conio, Francesco Bertuccio, Ilaria Giana, Lorenzo Arlando, Marianna Russo, Simone Montini, Chandra Bortolotto, Angelo Guido Corsico, Giulia Maria Stella

**Affiliations:** ^1^ Department of Internal Medicine and Medical Therapeutics, University of Pavia Medical School, Pavia, Italy; ^2^ Cardiothoracic and Vascular Department, Unit of Respiratory Diseases, Fondazione Istituto di Ricovero e Cura a carattere Scientifico (IRCCS) Policlinico San Matteo, Pavia, Italy; ^3^ Diagnostic Imaging and Radiotherapy Unit, Department of Clinical, Surgical, Diagnostic, and Pediatric Sciences, University of Pavia Medical School, Pavia, Italy; ^4^ Radiology Institute, Fondazione Istituto di Ricovero e Cura a carattere Scientifico (IRCCS) Policlinico San Matteo, Pavia, Italy

**Keywords:** cancer, microbiome, bronchiectasis, pre-metastatic niche, inflammation

## Abstract

The lungs are most often a preferential target organ for malignant spreading and growth. It is well known that chronic parenchymal inflammation and prolonged injuries represents an independent risk factor for cancer onset. Growing evidence supports the implication of lung microbiota in the pathogenesis of lung cancer. However, the full interplay between chronic inflammation, bacterial colonization, pathologic condition as bronchiectasis and malignant growth deserves better clarification. We here aim at presenting and analyzing original data and discussing the state-of-the-art on the knowledge regarding how this complex milieu acts on the plasticity of the lung pre-metastatic niche to point out the rationale for early diagnosis and therapeutic targeting.

## Highlights

- Due to the complex interplay between microenvironment, microbial coloniziation and chronic inflammation, bronchiectasis can act as risk factors for cancer onset and progression by priming the pre-metastatic niche.
- A more efficient multidisciplinary management of bronchiectasis should encompass oncologic screening for early cancer diagnosis and personalized therapeutic design.

## 1 Introduction

Non-cystic fibrosis bronchiectasis (NCFB) is a chronic airway disease defined by abnormal and permanent dilatation of the bronchial lumen. It presents with a clinical syndrome of cough and sputum production and is characterized by recurrent acute exacerbations and respiratory infections. This heterogeneous disorder, whose prevalence is higher in women and advanced age, can frequently occur post infection. Other causes may be inflammatory, allergic, autoimmune and immunodeficiency associated processes. Moreover, bronchiectasis can be of congenital and genetic origin or idiopathic (1, 2). The pathophysiology builds on chronic inflammation and is supported by a vicious circle of damage to the airways. Local defenses of bronchial wall, notably mucociliary clearance, are compromised by inflammation as a response to different stimuli, thus enhancing further infections and perpetuating inflammatory state (3, 4). The most frequent isolated microorganisms in the airways of patients with bronchiectasis are Gram-negative, like *Pseudomonas aeruginosa*, *Haemophilus influenzae* and *Moraxella catarrhalis*, but also *Staphylococcus aureus* and *Streptococcus pneumoniae* between Gram-positive. In addition, these patients are at an increased risk of nontuberculous mycobacteria (NTM) infection (5–8). Chronic *Pseudomonas aeruginosa* infection is recognized as a risk factor for adverse outcome and, there is speculation that the presence of different patterns of microbiome-inflammation interactions in patients with this chronic infection impact on exacerbations (9). However, there are still many questions about the mechanisms behind airway inflammation in bronchiectasis, such as the existence of different endotypes that are linked to different molecular pathways and can lead to customized treatment. Inflammation has a prominent role to subtype the disease in terms of clinical phenotypes and outcomes. The dominant inflammatory endotype is neutrophilic inflammation, featured by sputum purulence and tending towards bacterial load. Eosinophilic inflammation, Th2 mediated, has been observed in association with mucus plugging and asthma. The future implications of this are that dominant neutrophilic disease may respond to airway clearance, macrolides and log-term antibiotics, instead eosinophilic inflammation may benefit from inhaled corticosteroids (ICS) and innovative monoclonal antibody therapy (10, 11). At the present time, these topics are of high interest, together with microbiota and gene expression changes in airway epithelial cells, in order to identify an increasingly tailored therapeutic strategy. Lung cancer is the primary cause of cancer-related mortality globally for both men and women. Tobacco smoking is the most significant etiological factor for lung carcinogenesis, with the cumulative smoking exposure in pack-years serving as a critical metric for identifying individuals at high risk for developing LC and potentially benefiting from screening (12, 13). Other contributory risk factors include genetic susceptibility, occupational exposures, air pollution, and previous respiratory diseases (14). In COPD patients with chronic obstructive pulmonary disease (COPD), chronic inflammation leading to repeated airway injury and increased cellular turnover rates likely plays a pivotal role in lung carcinogenesis. Similarly, hypotheses linking previous lung infections to lung cancer suggest that inflammatory dysplasia caused by infections may progress to cancer (15). Consequently, NCFB should be hypothesized as a risk factor for LC (16).

## 2 Bronchiectasis and lung cancer: what we know from epidemiology and clinics

**Figure 1:**
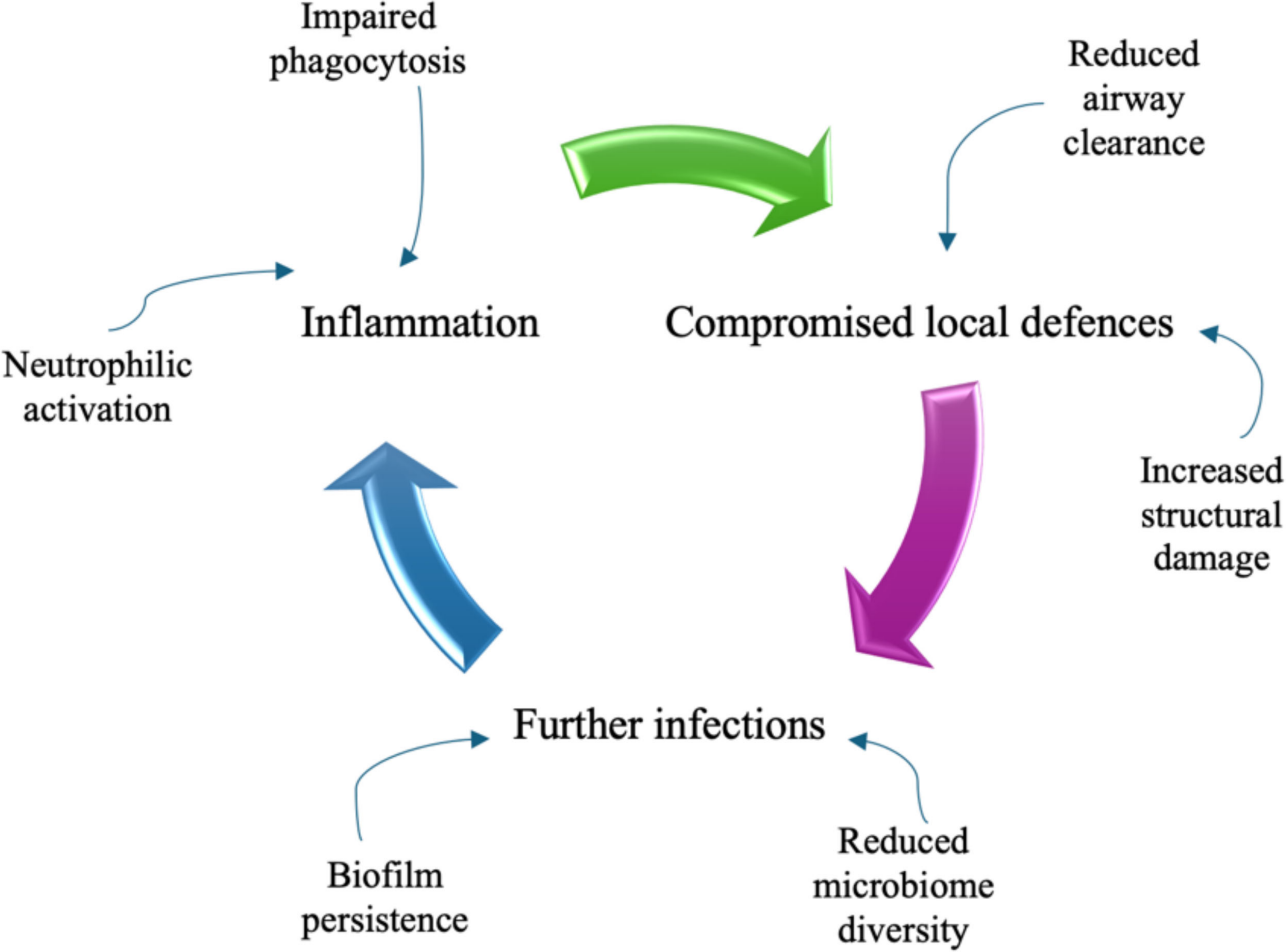
The vicious circle of damage to the airways in bronchiectasis.

It has long been hypothesized that there is a relationship between lung inflammation and lung cancer because of the repeated airway injury and its consequent high cell turnover. Nevertheless, this aspect has been effectively investigated mainly in chronic obstructive pulmonary disease (COPD) and the evidence for this mechanism remains weak and somehow unclear, due to the scarcity of mechanistic studies evaluating the association between lung cancer (LC) and chronic inflammatory lung diseases other than COPD (17–19). On the other hand, bronchiectasis is characterized by the enlargement of bronchial tubes, due to an inflammatory injury that undermines protective mechanisms of airway walls, by exposing them to further damage (**Figure 1**).

Cytokines and chemotactic factors, such as interleukin 8 (IL-8 or CXCL-8), interleukin 1 beta (IL-1), interleukin 17 (IL-17), tumour necrosis factor alfa (TNF) and leukotriene B4, attract neutrophils from the blood to the airways, where they are activated and can produce reactive oxygen species (ROS), release granule products (myeloperoxidase, neutrophil elastase, heparin-binding protein, resistin and matrix metalloproteinases) and form neutrophil extracellular traps (NETs).

The latter are webs of DNA, histone proteins and neutrophil proteases, the role of which is to trap and kill microbes; however, their overabundance is involved in the progression of bronchiectasis and chronicity of infection, along with impaired neutrophil phagocytosis and disrupted mucociliary clearance (1, 20–22).

Moreover, the clearance of apoptotic cells by macrophages, known as efferocytosis, is impaired in bronchiectasis and is linked to an increase in inflammation and airway damage, by promoting secondary necrosis. And by the way, lung macrophages may induce an infiltration of neutrophils via TNF-α production (1, 20–22).

In bronchiectasis, any of these pathways can be affected, as evidenced by decreased pathogen clearance and structural damage, linked to an overabundance of neutrophil elastase, which can cleave and inactivate host proteins including cell receptors involved in efferocytosis, antimicrobial peptides and extracellular matrix proteins. In addition, bacterial biofilm can be paradoxically stabilized by neutrophils because it utilizes extracellular DNA released from neutrophils and protects bacteria from the host immune system hindering phagocytosis (1, 20–22).

Most of the mechanisms of airway inflammation in bronchiectasis remains unclear and is still under study. On this basis, given inflammatory state is implicated in carcinogenesis, it begs the question of a possible correlation between bronchiectasis and LC. Several authors were interested in this inquest, obtaining mixed results (23–26). A recent Italian systematic review (27) founds that bronchiectasis are associated with a higher risk of developing LC and that this risk is higher for males, the elderly, and smokers, whereas the effect of concomitant COPD is unclear. Similarly, Chung et al. conducted a longitudinal nationwide cohort study in Taiwan. According to their findings, patients with bronchiectasis exhibit an increased risk of LC compared with the general population (28). Identifying risk factors and developing predictive scores are essential for determining patients who may benefit from LC screening, which can be integrated into clinical practice. McDonnel and colleagues recently developed the Bronchiectasis Etiology Comorbidity Index (BACI) from a European multicenter cohort to identify comorbidities associated with mortality risk in patients with bronchiectasis (29). Their study demonstrated a positive association between bronchiectasis and malignancies, including lung cancer, highlighting the need for more detailed data on factors associated with LC risk in these patients. However, the presence of confounding factors in the analysis of this topic is not negligible. Since smoking is one of the major factors that increase the incidence of LC controlling for smoking is essential for evaluating this causal relationship with chronic lung disease. Therefore, COPD is another confounding factor in the analysis of the association between bronchiectasis and LC. Interestingly, some Korean works have paid attention to this aspect. Results of a matched case-control study conducted at Seoul National University Boramae Medical Center (30) showed that bronchiectasis was associated with a lower risk of LC, assessing by histology (significant for squamous cell carcinoma) and smoking status, in COPD patients with moderate to very severe airflow limitation. Another population-based study, using the Korean National Health Insurance Service (NHIS) database from 2009 to 2018 or until the date of incident lung cancer/death, noted an overall incidence of LC in bronchiectasis cohort of 1.9% with a significant higher risk for male sex, overweight, current smoking, living in rural areas, and comorbid COPD (31). A similar population-based cohort study conducted by Choi et al. on the Korean NHIS found that patients with bronchiectasis had a significantly increased risk of developing LC compared with those without bronchiectasis and this association was significant in patients over 60 years of age (32). In addition, two interesting data emerged. A significant interaction between smoking status and bronchiectasis have not been detected in increasing LC risk. Furthermore, despite bronchiectasis having a significant impact on the LC risk in individuals without COPD, it did not affect the risk of LC in those with COPD. Concerning the smoke, in contrast, Sin et al. found a significant interaction between bronchiectasis and smoking regarding the risk of LC-related mortality (33). The Korean group also did a multi-center retrospective study (34), analyzing the CT appearance of LC and bronchiectasis as event variables on lung lobes. They revealed that pre-existing bronchiectasis was associated with a significantly lower risk of LC in the same lobe, suggesting that chronic inflammation involved in bronchiectasis might produce different cytokines and acts in a different way in carcinogenesis of the lung. In an interesting way, in a LC screening study conducted by Sanchez-Carpintero Abad et al. (35), using Pamplona (Clìnica Universidad de Navarra, Spain) sub-cohort of the International Early Lung Cancer Action Program (I-ELCAP) between 2000 to 2012, the prevalence of bronchiectasis in smokers (most patients had mild bronchiectasis and were asymptomatic) was high and had an impact on the need for additional tests, but not on the incidence of cancer. Results of the study showed that having bronchiectasis increases the probability of finding nodules on baseline low dose computed tomography (LDCT), conditioning the workup of benign nodules and having an impact on costs. By reaching the most complex study part, increasing interest is being shown in the potential molecular mechanisms involved. In LC associated to bronchiectasis the microorganism balance is disturbed, and it was thought that the microbiota would to play a significant role in disease causation and progression. Recent studies focus on this point, stimulating the use of genetic investigations. Ekanayake et al. have researched bacteria in oropharyngeal swab and bronchoalveolar lavage of two disease groups of patients with LC and bronchiectasis, using 16S rRNA gene-based metagenomics and basic bacterial culturing (36). Also relevant is that Metagenomic Next-Generation Sequencing (mNGS) shortens the turnaround time and shows to be a valuable tool for bronchiectasis pathogen detection (37). Currently, a particular focus is molecular profiling of the airway epithelium in bronchiectasis, indeed several studies have identified gene expression alterations. Decreased protocadherin gene expression affects cell adhesion and, as noted by Xu et al., reduced expression in the Wnt signaling pathway in basal cells compromises the epithelial niche and the balance of epithelial/mesenchymal interactions (38). In addition, increased expression of genes involved in ciliogenesis, as a response to ciliary damage, may result in overproduction of certain ciliary proteins that can lead to defective cilia assembly. In this study, conducted on individuals with radiological bronchiectasis but without a clinical diagnosis, is also recognized the potential role of proteasome-related proteins in the inflammatory process (upregulation of constitutively expressed proteasome 20S subunit-β and IFN-γ inducible immunoproteasome subunits). The open question is whether these bronchial gene expression changes in bronchiectasis may be involved in the development or progression of LC. Do not overlook the data coming from few studies that reports elevated serum TGF-β1 (Transforming Growth Factor-β1) in patient with bronchiectasis. TGF-β1 is known to protect against carcinogenesis by regulating cellular proliferation, differentiations, survival, but also adhesion and cellular microenvironment. In addition, the ΔF508 deletion in *CFTR* gene in patients with cystic fibrosis, which shows radiological bronchiectasis, is inversely associated with malignancies. All this data is the starting point for the investigation of a disease that is more widely recognized and its potential impact on LC, resulting in the constantly evolving management of bronchiectasis. This could have significant consequences for the management of follow-up and treatment. There are several open points related to searching for customized targets on pathology endotypes, but the lapel can also trivially affect the control of the inflammatory state and the widely debated use of corticosteroids.

## 3 The role of mucins and their glycosylation in the respiratory tract infections

**Figure 2:**
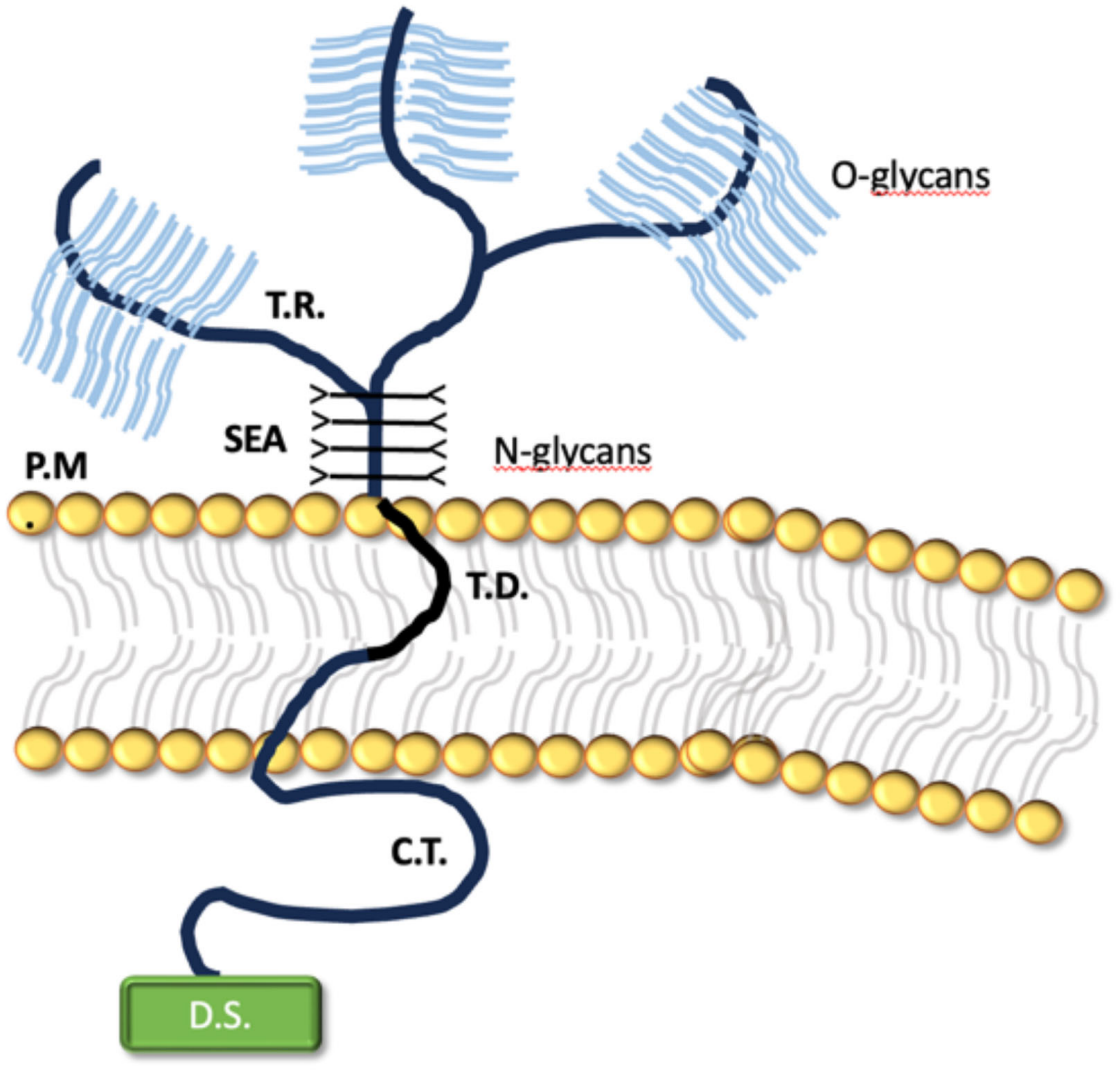
The transmembrane structure of MUC1.

Mucins are large glycosylated glycoproteins that have a fundamental role in protecting mucosal surfaces throughout the body. Due to their structure, by providing ligands for pathogen binding and the ability to shed the bound extracellular domain, mucins can act as a releasable decoy barrier to mucosal pathogens. They can also sterically block binding to underlying cellular receptors. The cytoplasmic tail domain is capable of initiating signal transduction cascades and due to their conservation across species, may play an important biological role in cellular signaling. MUC1 is one of the most extensively studied of the cs-mucin family (**Figure 2**). It has been demonstrated to play a dynamic role in protection of the host from infection and to regulate inflammatory responses to infection. It has also been studied for its aberrant expression and role in cancer (39). It doesn’t only provide a physical barrier, limiting infection and colonization, but it also plays a significant role as a modulator of pathogen-induced inflammation (40–42). The cytoplasmic tail (-CT) of MUC1 which is conserved throughout most species, possesses seven tyrosine residues, 4 of which can be phosphorylated by kinases and initiate signal transduction cascades. The presence of extracellular EGF-like domains and phosphorylation sites in the cytoplasmic tail suggest that MUC1 is able to have a functional role in signaling cascades, enabling recycling of the MUC1-ED for re-glycosylation after degradation in the lumen, or as a result of microbial interaction (43, 44).

It encompasses a large, extracellular, O-glycosylated backbone defined by variable sequences of tandem repeats (T.R). The juxtamembrane portion of the protein is made by the sea-urchin sperm protein, enterokinase and agrin (SEA) domain, linked to N-glicans. MUC1 transmembrane domain (T.D.) is made of 28 amino acids and binds the protein across the plasma membrane (P.M.) of the cell. Notably, the protein cytoplasmatic tail (C.T.) displays a multifunctional docking sites (D.S.) which allows intercation with kinases and other proteins.

In respiratory tract infections, MUC1 plays a crucial role in controlling inflammatory responses. It acts as an adhesion site for the flagellin of *Pseudomonas aeruginosa* (45). Research has shown that mice lacking MUC1 (Muc1−/−) reduced the retention time of *P. aeruginosa* in their lungs compared to wild-type (WT) mice. In a murine model of *P. aeruginosa* infection,in MUC1 deficient mice the reduced colonization was associated with better pathogen clearance due to a stronger early infiammatory response compared to WT mice (46). Interestingly, the host enzyme Neuraminidase 1 (NEU1) affects the ability of MUC1 to protect against P.aeruginosa. The flagellin from this bacter activates NEU1, which desialates MUC1 and other cell surface receptors, making it easier for P.Aeruginosa to bind and increase infection risk (47). Infections by *P. aeruginosa* and respiratory syncytial virus activate Toll-like receptors (TLRs) in airway epithelial cells and macrophages, leading to the production of inflammatory mediators like IL-8 and TNFα, which recruit immune cells to the site of infection (48). In response, MUC1 is upregulated and acts to suppress TLR signaling, thereby reducing inflammation. While the exact timing and degree to which MUC1 inhibits TLR pathways to reduce inflammatory responses remain unclear, this process is thought to be critical in controlling the severity of infection.

MUC1 also plays a role in *Streptococcus pneumoniae* infections. The bacterium’s ability to bind to epithelial cells is MUC1-dependent, and their interaction may trigger phagocytosis. MUC1-deficient macrophages have been shown to be inefficient at phagocytosing the pneumococci (49).

In the case of Influenza A virus (IAV), MUC1 has been demonstrated to bind to viral particles due to its sialylated structure, potentially reducing the virus’s ability to infect host cells. Elevated MUC1 levels are seen in various respiratory conditions, suggesting a functional role in these disorders. The sialylated carbohydrate antigen KL-6, identified as human MUC1, serves as a biomarker for interstitial lung disease. Additionally, increased MUC1 levels have been found in patients with cystic fibrosis, severe pneumonia, asthma exacerbations, measles pneumonia, and COPD.

Infection-induced cancers are the fourth leading cause of cancer-related deaths globally. Microbes interact with the host through glycosylated mucin proteins forming a protective barrier. Mucin protein MUC1, a key regulator of NF-κB, plays a protective role during microbial invasion by reducing inflammatory responses. However, prolonged microbial interaction can alter MUC1 glycosylation, compromising the epithelial barrier and shifting MUC1’s role from anti-inflammatory to proinflammatory, enhancing oncogenic signaling through its cytoplasmic tail (50, 51).

In bronchiectasis, mucins and their glycosylation play a crucial role in both protecting the airways and disease progression. Altered glycosylation of mucins can lead to bacterial colonization, chronic infections, and imbalances in the lung microbiome, which further exacerbates respiratory complications. In bronchiectasis, excessive mucus production and abnormal mucin glycosylation can impair the clearance mechanism. This dysfunction allows bacteria to colonize the lungs more easily, leading to persistent infections and chronic airway inflammation, further aggravating the disease (52, 53).

## 4 The lung hypoxia, microbes and pre-metastatic niche: a dynamic milieu

The tumor microenvironment is made up of stromal (immune cells, fibroblasts and endothelial cells) and cancer cells, extracellular matrix and different mediators released in the tumor site. Metabolic changes, hypoxia, and infiltration of immunosuppressive cells (myeloid-derived suppressor cells, tumor-associated macrophages, tumor-associated neutrophils and regulatory T cells) are all possible factors that can influence the resistance and progression of malignant cells in this intertwined network (54, 55). Besides, anticancer drug resistance seems to be affected by intracellular pathogens in the tumor microenvironment because bacteria can metabolize chemotherapeutic drugs and alter cytotoxicity, as well as acting as reservoirs in the development of metastasis (56). Between immunosuppressive cells, great attention is given to macrophages, which are distinguished in anti-tumoral (M1-MQ, that produce pro-inflammatory cytokines such as tumor necrosis factor-alpha [TNF-α], interferon-γ [IFN-γ] and interleukin-12) and pro-tumoral (M2-MQ, involved in hemostasis, wound-healing and tissue remodeling phenomena) populations in the tumor microenvironment, where hypoxia leads to MQ polarization toward the M2 phenotype. This cell population play a key role in different phases of tumorigenesis, including immunosuppression, angiogenesis and metastasis (57). Hypoxia may also influence gene expression profiles, like the upregulation of hypoxia-responsive transcription factors (hypoxia-inducible factor (HIF)− 1α as the main) in macrophages. HIFs are implicated in various mechanisms promoting cancer progression: angiogenesis, epithelial-mesenchymal transition, cell motility, metabolic reprogramming, extracellular matrix, immune evasion and cancer stem cell specification (58–61). Moreover, hypoxia can trigger a type of programmed cell death, associated closely with inflammatory responses, called pyroptosis (62). This is a secondary necrosis, where apoptotic cells are not promptly removed by phagocytosis and instead go on to display necrotic cell death with the release of intracellular contents. This process may elicit adaptive immune responses and, at the same time, it is primarily initiated by the induction of inflammation (63). Metastatic progression defines the aggressivity and high malignancy of neoplastic diseases. Distant spreading of cancer cells is orchestrated by a series of biologic processes which encompass a complex interplay between genetic drivers and immune-inflammatory and hypoxic settings (64–67). This dynamic dialogue is the basis not only for the gain by malignant cells of those features and properties which are required to leave the niche (68, 69), but also determine the site/organ where they will stop and will give rise to secondary masses (70–72). Since the Paget’s hypothesis of “seed and soil” (73), the concept that tumor surrounding stroma plays a role in disease progression has been widely expanded ultimately leading to the development of immunotherapy against cancer (74–76). In this context, the primary lesion can actively select and modify the microenvironment of distant sites to facilitate and support metastatic growth. Circulating tumor cells (CTCs) cooperating with biochemical mediators, growth factors and exosomes secreted by the primary tumor assure disease progression by interacting with the pre-metastatic niche (77). The latter is, thus, characterized by the appearance of the following features: increased vascular permeability, extracellular matrix remodeling, bone marrow-derived cells recruitment, angiogenesis, and immunosuppression (78); namely those features defining the epithelial-to-mesenchymal transition process (79). Notably, inflammatory cells such as neutrophil extracellular traps (NETs) and proteins as metalloproteinases can play a role in awakening dormant cancer cells to acquire invasive phenotype (80). On these premises, a recent amount of data points out the role of microbiota and microbiome during cancer onset and progression. We and others have reported that microbiota plays a role not only in cancer disease predisposition and risk but also in its initiation and progression, with an impact on patients’ prognosis (81). The question regarding the role of microbes in cancer cell invasion capacity is still a debated issue. The local microbiota can affect migration and motility of primary tumor cells (82–84), as shown in colorectal (85–88), breast (89), head and neck (90–94), pancreatic (95), prostate (96), bladder (97) cancer and melanoma (98). Within respect to the lung cancer, it is well known that although the NSCLC patients had similar microbial communities with non-cancer controls, rare species such as Lactobacillus rossiae, Bacteroides pyogenes, *Paenibacillus odorifer, Pseudomonas entomophila, Magnetospirillum gryphiswaldense*, fungus *Chaetomium globosum* et al. showed obvious difference between NSCLC patients and non-cancer controls, namely defining a dysbiosis condition (99, 100). Notably, a specific association between microbial species and cancer patient gender and smoking habit has been reported (101) and species in NSCLC patients are also associated with gene expression profile as reported for the *EGFR* status (102). Thus, a certain impact microbes of on cancer progression is documented, although fewer data are available on how lung chronic infection could affect metastatic phenotype and contribute to the construction of the pre-metastatic niche.

## 5 Personal insights

The lung can be the site of growth of secondary lesions [^Kolling^] and of primary massese and the effect of dysbiosis and/or infections and colonization should be investigated under these two perspectives. During lung infection, it has been shown that bacterial endotoxins, through endotoxin receptor TLR4 that is expressed in endothelial cells and leukocytes, act by increasing immuno-inflammatory reactions characterized by increasing vascular permeability and leukocyte mobilization (103). The primary tumor can act on innate immune reaction which, in turn cooperate in priming the pre-metastatic niche (104). Indeed, cancer cells produce chemokine CCL2 a potent chemoattractant for monocytes, macrophages, memory T lymphocytes, and natural killer (NK) cells which is known to be implicated in cancer progression (105–108). CCL2 derives from hypoxic primary cancer and is associated to immunosuppression which characterizes the lung premetastatic niche by promoting the infiltration of dysfunctional myeloid and NK cells with decreased capacity to eliminate incoming invasive tumor cells (109). Moreover, CCL2 by paracrine signal, can induce lung overexpression of endogenous TLR4 ligands such as the myeloid cell-derived proteins S100A8 and SAA3 (110–113). Tumor-derived extravesicles are also implicated in suppression of T cells in the niche through a link with cancer-associated fibroblasts (114–116). Indeed, they can secrete CCL1, a chemokine which is involved in inflammatory diseases (117), which induced Treg differentiation by activating its specific receptor CCR8, ultimately contributing to the establishment of an immunologically tolerant niche (118, 119). On the other hand, in some instances bacteria can prepare the niche since they can favour tumor distant spreading by acting on vascular permeability. It has been reported in colorectal cancer (CRC) where resident bacteria *Escherichia coli* can disrupt the gut vascular barrier thus facilitating liver metastatization, as shown by the increase of plasmalemma vesicle-associated protein-1 (PV-1) which is a protein associated to endothelial fenestration (120–122). Even dietary factors, as capsaicin, can increase barrier permeability by facilitating proliferation of e of mucin-related bacteria like *Akkermanisa* and *Muribaculaceae* and bacteria involved in bile acids metabolism, whose alterations are implicated in the recruitment of NK cells in the pre-metastatic niche (123). Within respect to the pathogens that are frequently associated to bronchiectasis, causative relationship between microbial infection and cancerogenesis is still controversial. Chronic infection by NTBCs, as *Mycobacterium Avium Complex* (MAC), is known to be implicated in different steps of lung carcinogenesis (124). MAC infection has been reported to be associated to the arousal of squamous cell tumors, mainly localized in peripheral lung parenchyma (overlying bacterial infection in distal airways) and mainly effecting non-smoker women (125). Indeed pathogen-induced inflammation activates proliferative pathways in machrophages and epithelial cells through the nuclear factor NF-κB, direct DNA damage and enhances expression of cyclooxygenase-2 and ultimately promotes tumor angiogenesis (126). A different mechanism sustains the crosstalk between *Pseudomonas aeruginosa* and cancer since it has been reported an antiproliferative effect induced by upregulation of the Pseudomonas-secreted cupredoxin azurin, according to a dual relationship between tumor and bacteria, based on the modulation of secretion of aldolase A in response to contact with azurin. Coherently, cancer patients with concomitant detection of *P. aeruginosa* display an increased overall survival (127, 128).

In the cohort of 119 patients affected by NCFB diagnosed (mainly of idiopathic origin) and followed in our Institution (Unit of Respiratory Diseases Fondazione IRCCS Policlinico San Matteo Pavia) in the last year, the percentage of subsequent diagnosis of solid cancer is of 17.5% (19 cases). Interestingly none of the patients developed primary lung cancer, being over 73% of patients past or never smokers. The arousal of cancer occurred in patients featuring moderate/severe bronchiectasis, with frequent exacerbation rates. Demographic and clinical features of the cohort analyzed and infection/colonization history and tumor data are reported in **Tables 1** and **2** respectively.

**Table 1:** Clinical and demographic data of the cohort of patients, affected by NCFB who developed cancer, in care of the Unit of Respiratory Diseases Fondazione IRCCS Policlinico San Matteo Pavia in the last year.

**GENDER**	F				M			
	15				4			
**SMOKE**	Never		Current				Past	
	11		4				3	
**BMI < 18**	3							
**MEDIAN CMI**	3.7							
**COMORBIDITY**	COPD	Asthma		GERD		Cardiopathy		Rheumatic
	4	1		7		9		3
**MEDIAN AGE AT NCFB DIAGNOSIS (AGE)**	68.6							
**ORIGIN OF BRONCHIECTASIS**	Idiopathic				Post infective			
	12				4			
**TYPE OF BRONCHIECTASIS**	Cylindric		Cystic				Varicose	
	19		0				0	
**BSI**	Mild		Moderate				Severe	
	4		7				8	
**EXACERBATIONS**	Frequent				Free/rare			
	11				8			
**SITE OF CANCER ORIGIN**	Skin/melanoma		Breast				Bladder	
	6		9				4	

BSI, bronchiectasis severity index; CMI, Charlson comorbidity index; BMI, body mass index; COPD, chronic obstructive pulmonary disease; GERD, gastro-esophageal reflux disease.

**Table 2:** Data regarding microbial infection and colonization of the patients enrolled.

AETIOLOGICAL AGENT OF EXACERBATION	
*Streptococcus spp*	1
*Haemophilus influenzae*	5
*Staphylococcus aureus*	1
*Aspergillus fumigatus*	3
*Nocardia*	1
*Moraxella catarrhalis*	1
*Achromobacter spp*	1
NTM	3
SARS CoV-2	4

COLONIZATION	
*Pseudomonas* spp.	2
*Staphylococcus. aureus*	1
*Aspergillus* spp.	1
NTM	3
More than one pathogen	2

Overall, these observations might allow some clinical considerations. Bronchiectasis can act as risk factors for cancer onset and progression by acting on modulating the pre-metastatic niche. It is conceivable that infection and/or chronic colonization patterns could impact on the site of tumor growth by modulating invasive potential of transformed cells. Recommendations for oncologic screening for bronchiectasis patients should be underlined in the clinical context.

## Data Availability

The original contributions presented in the study are included in the article/Supplementary Material. Further inquiries can be directed to the corresponding author.
